# Regulation of Chitin-Dependent Growth and Natural Competence in *Vibrio parahaemolyticus*

**DOI:** 10.3390/microorganisms8091303

**Published:** 2020-08-26

**Authors:** Anusuya Debnath, Tamaki Mizuno, Shin-ichi Miyoshi

**Affiliations:** Graduate School of Medicine, Dentistry and Pharmaceutical Sciences, Okayama University, 1-1-1, Tsushima-naka, Kita-ku, Okayama 700-8530, Japan; mizuno-t@cc.okayama-u.ac.jp (T.M.); miyos-s@okayama-u.ac.jp (S.-i.M.)

**Keywords:** chitin, chitinase, GlcNAc_6_, natural competence, ChiA2, ChiS, TfoS

## Abstract

Vibrios can degrade chitin surfaces to soluble N-acetyl glucosamine oligosaccharides (GlcNAc_n_) that can be utilized as a carbon source and also induce a state of natural genetic competence. In this study, we characterized chitin-dependent growth and natural competence in *Vibrio parahaemolyticus* and its regulation. We found that growth on chitin was regulated through chitin sensors ChiS (sensor histidine kinase) and TfoS (transmembrane transcriptional regulator) by predominantly controlling the expression of chitinase VPA0055 (ChiA2) in a TfoX-dependent manner. The reduced growth of Δ*chiA2*, Δ*chiS* and Δ*tfoS* mutants highlighted the critical role played by ChiA2 in chitin breakdown. This growth defect of Δ*chiA2* mutant could be recovered when chitin oligosaccharides GlcNAc_2_ or GlcNAc_6_ were supplied instead of chitin. The Δ*tfoS* mutant was also able to grow on GlcNAc_2_ but the Δ*chiS* mutant could not, which indicates that GlcNAc_2_ catabolic operon is dependent on ChiS and independent of TfoS. However, the Δ*tfoS* mutant was unable to utilize GlcNAc_6_ because the periplasmic enzymes required for the breakdown of GlcNAc_6_ were found to be downregulated at the mRNA level. We also showed that natural competence can be induced only by GlcNAc_6_, not GlcNAc_2_, because the expression of competence genes was significantly higher in the presence of GlcNAc_6_ compared to GlcNAc_2_. Moreover, this might be an indication that GlcNAc_2_ and GlcNAc_6_ were detected by different receptors. Therefore, we speculate that GlcNAc_2_-dependent activation of ChiS and GlcNAc_6_-dependent activation of TfoS might be crucial for the induction of natural competence in *V. parahaemolyticus* through the upregulation of the master competence regulator TfoX.

## 1. Introduction

*Vibrio parahaemolyticus* is responsible for food-borne gastroenteritis globally since isolation of the first pandemic O3:K6 strains in 1996 [1]. This Gram-negative, halophilic bacterium is widely disseminated in estuarine, marine and coastal surroundings either in a free-swimming state or affixed to abiotic and biotic surfaces including zooplankton, fish and shellfish [2,3]. The most abundant biomolecule in this habitat is the insoluble polysaccharide known as chitin, composed of *β*→1,4 linked N-acetyl glucosamine (GlcNAc) residues. Through the action of secreted chitinase, Vibrios can degrade the chitin surfaces into soluble GlcNAc_n_ oligosaccharides and utilize them as a source of carbon and nitrogen [4]. Thus, *Vibrio* species in the aquatic environment are the key players in the recycling of chitin [5]. According to the *Vibrio cholerae* chitin utilization pathway, it is known that the chitinase enzymes degrade chitin into GlcNAc_2-6_ oligosaccharides which then enter either through porin or chitoporin, depending on their sizes. Then, further enzymatic degradation takes place in the periplasm to primarily release GlcNAc_2_ from higher oligosaccharides along with some GlcNAc residues [6,7]. The binding of GlcNAc_2_ with CBP (chitin oligosaccharide binding protein) activates ChiS (sensor histidine kinase) which is the regulator of genes required for degradation and utilization of chitin, such as chitinases for chitin breakdown, chitoporin for transport of GlcNAc_n_ residues into the periplasm and GlcNAc_2_ catabolic operon to metabolize GlcNAc_2_ in cytoplasm [4,8].

In addition to its role as a nutrient source, chitin can also activate a cascade of gene expression to induce natural competence in Vibrios. Natural competence is the ability to uptake extracellular DNA (eDNA) from the environment and this eDNA might get integrated by homologous recombination to provide novel traits. Chitin-induced natural competence and uptake of eDNA was first reported in *V. cholerae* in 2005 [9]. Thereafter, it was observed in several species of the Vibrionaceae family such as *Vibrio vulnificus*, *Vibrio fischeri* and *V. parahaemolyticus* [10,11,12]. This process of horizontal gene transfer is known as natural transformation and is one of the reasons behind the high levels of genomic diversity among Vibrionaceae [9,10,11,12]. In *V. cholerae*, the competence for genetic transformation is triggered by chitin-induced transcription factor TfoX, which regulates the genes required for DNA uptake [9]. The exposure of chitin oligosaccharides induces the transcription of Hfq-dependent small RNA (sRNA), *tfoR,* which is critical for the translation of TfoX [13]. Recently, two independent studies characterized a novel chitin-sensing regulator, TfoS, that is responsible for transcriptional activation of *tfoR* [14,15]. Yamamoto et al. reported that TfoS does not possess the signature domain of a two-component system (TCS), but the activity of TfoS is dependent on ChiS.

Due to the conserved nature of chitin utilization and natural transformation among Vibrios, these two processes were primarily studied in *V. cholerae*. However, a recent report showed the existence of variability in the regulation of natural transformation among Vibrio species [16]. So, we used *V. parahaemolyticus* in our study to determine the role of chitinases, ChiS and TfoS in terms of chitin utilization and uptake of eDNA. We also studied the effect of GlcNAc_2_ and GlcNAc_6_ on the natural competence of *V. parahaemolyticus* which will highlight a new aspect of regulation related to this conserved phenomenon.

## 2. Materials and Methods

### 2.1. Bacterial Strains, Plasmids Used and Growth Conditions

*V. parahaemolyticus* strain RIMD2210633 (Wild-type; WT), an O3:K6 serotype [1] was obtained from the Laboratory for Culture Collection, Research Institute for Microbial Diseases, Osaka University. This strain was used for the construction of deletion mutants and for functional studies. The WT strain was grown aerobically (250 rpm) at 30 °C for 16 h in 5 mL marine Luria–Bertani (MLB) broth (LB broth containing 3% NaCl). For this study, we had generated a spontaneous streptomycin-resistant (SmR) mutant of the WT strain as described previously [17] and designated as VPS. The frequency at which we isolated SmR mutant was 1 × 10^−10^. The genomic DNA (gDNA) isolated from VPS was used in chitin-induced natural transformation assays.

The isogenic deletion mutants were constructed by double-crossover allelic exchange using the R6K-*ori* suicide vector pXAC623 [18] and was maintained in *Escherichia coli* β2155 *λ* pir, a diaminopimelic acid (DAP) auxotrophic mutant [19]. For TA cloning, we used pGEMT easy vector (Promega, Madison, WI, USA) and was maintained in *E. coli* JM109. *E. coli* strains JM109 and β2155 were routinely cultured in LB broth at 37 °C. However, 0.5 mM DAP (Wako, Osaka, Japan) was added for the growth of *E. coli* β2155. The medium was also supplemented with appropriate antibiotics whenever necessary.

### 2.2. Construction of Isogenic Deletion Mutants of V. parahaemolyticus RIMD2210633

All the in-frame deletion mutants (Table 1) were created using splicing by overlap extension (SOE) PCR and allelic exchange [20]. Primers were designed using the *V. parahaemolyticus* RIMD2210633 genome sequence [21] as the template. For each gene deletion, approximately 500–600 bps flanking sequence of the genes (*vp2478*: *chiS*; *vpa1177: chiA*; *vpa0055: chiA2 and vp0854: tfoS*) were amplified using two sets of primers. These upstream and downstream flanking PCR products were then fused by PCR to get an in-frame truncated version of the respective gene. This fusion product was amplified and cloned into pGEM-T Easy vector. This plasmid was then digested with a pair of restriction enzymes and the insert was then ligated into the suicide vector pXAC623. The resulting plasmid was transformed into *E. coli* β2155 *λ* pir (donor) and then mobilized into WT strain (recipient) by filter mating. In brief, the donor and the recipient strains were grown until the OD_600_ reached 0.4–0.5. Then, the donor and recipient strains were mixed in a 1:1 ratio and spotted upon 0.22 μm filter membrane (Millipore, Burlington, MA, USA) placed on LB plates and kept at 30 °C for overnight. The transconjugants were selected by the absence of DAP and presence of chloramphenicol in the selection plates. These colonies were streaked on MLB plates with 10% sucrose without chloramphenicol and incubated at 30 °C for overnight to select for colonies with desired gene deletion. Double-crossover deletion mutants were screened by PCR based assay using specific primers.

### 2.3. Growth Curve Analysis

From overnight grown cultures, fresh MLB broth was inoculated in 1:100 dilution and grown until log phase was reached. Then the log phase cultures were harvested by centrifugation and washed with defined artificial sea water, DASW [9]. 30 mL of DASW supplemented with 1% shrimp shell chitin flakes (Sigma-Aldrich, St. Louis, MO, USA) was inoculated with approximately 10^4^ cfu mL^−1^. The cultures were incubated at 30 °C under shaking of 100 rpm for 72 h. In case of chitin oligosaccharides, 5 mM GlcNAc_2_ and 1.25 mM GlcNAc_6_ supplemented DASW was used and growth was checked for 48 h at 30 °C.

### 2.4. Chitinase Plate Assay

Colloidal chitin was prepared from chitin flakes derived from shrimp shells as previously described [22]. Colloidal chitin plates were made by mixing 2% *w*/*v* colloidal chitin in DASW. Strains were grown in MLB broth at 30 °C until OD_600_ reached 0.4. Then, the cell suspension was washed twice with DASW and diluted in DASW so that the OD_600_ became ~0.4. 10 μL of each bacterial suspension was spotted on the chitin agar plate. The plate was incubated at 30 °C for 5 days and the zone of chitin clearing was recorded.

### 2.5. Natural Transformation Assays in the Presence of Chitin Flakes and Purified Chitin Oligosaccharides

Natural transformation assays were performed as previously described [23] with some modifications. WT and isogenic mutant strains were inoculated in MLB broth for overnight growth. The overnight grown culture was diluted with fresh MLB broth in a 1:100 ratio and grown until the OD_600_ reached 0.3–0.4. The bacterial pellet was then washed twice with DASW and diluted in DASW + 0.2% lactate so that the OD_600_ became ~0.2. This 4 mL of bacterial suspension was added to the conical flask having 200 mg sterilized chitin flakes and incubated statically at 30 °C for overnight. Next day, planktonic phase was removed and fresh 4 mL DASW added along with 50 μg of gDNA (conc. 12.5 μg mL^−1^) isolated from streptomycin resistant strain VPS and incubated at 30 °C for 8 h under static condition. In negative control only TE buffer was added. After 8 h incubation period, the conical flask was vigorously vortexed to release the attached bacteria. The appropriate dilutions were then plated onto MLB plates with or without streptomycin (200 μg mL^−1^). The transformation efficiency was calculated as the number of colonies in antibiotic plates divided by the number of colonies on plates without antibiotic.

To determine the frequency of transformation in liquid culture, cells were grown to late-log phase in DASW supplemented with different chitin oligosaccharides (Carbosynth Limited, Berkshire, UK) in the form of GlcNAc_2_ (5 mM) and GlcNAc_6_ (1.25 mM). Approximately, 12.5 μg mL^−1^ genomic DNA was added and incubated at 30 °C for 8 h under static condition. In negative control only TE buffer was added. The appropriate dilutions were then plated onto MLB plates with or without streptomycin (200 μg mL^−1^).

### 2.6. Total RNA Isolation and Real-Time PCR

WT and isogenic mutant strains were inoculated in MLB broth for overnight growth. Overnight grown culture was added to fresh MLB broth in 1:100 dilution until the OD_600_ reached 0.3–0.4. The bacterial pellet was then washed twice and diluted in DASW + 0.2% lactate so that the OD_600_ became ~0.2. This 5 mL of bacterial suspension was added to the conical flask having 100 mg sterilized chitin flakes and incubated statically at 30 °C for 24 h. Then, the conical flask was vigorously vortexed to release the attached bacteria and the complete bacterial suspension was pellet down. Next, this bacterial pellet was dissolved in 500 μL PBS and 1 mL RNAprotect solution and incubated for 5 min at room temperature. The RNA extraction was done according to manufacturer protocol by using RNeasy kits (QIAGEN, Hilden, Germany). The enzymatic lysis of *V. parahaemolyticus* was performed with 200 μL of lysozyme (5 mg mL^−1^) for 15 min at room temperature. In-column DNase treatment was performed for 15 min at room temperature.

For the mRNA expression analysis in the presence of purified chitin oligosaccharides, we used 2.5 mM GlcNAc_2_ and 0.625 mM GlcNAc_6_. Overnight grown culture was added to fresh MLB broth in 1:100 dilution until the OD_600_ reached 0.3. The bacterial pellet was then washed twice and diluted in DASW + 0.2% lactate + GlcNAc_n_ so that the OD_600_ became ~0.2. This bacterial suspension was incubated at 30 °C for 5 h and then processed for RNA isolation. The primers were designed by Primer Quest tool of Integrated DNA Technologies (IDT, Coralville, IA, USA) such that the amplicon size should be approximately 80–165 bp. We used iTaq Universal SYBR Green One-Step kit for real time RT-PCR (Bio-Rad*,* Hercules, CA, USA) with 50 ng of RNA for each reaction in Mini Opticon Real-time PCR system. The relative expression of the target transcripts was calculated according to Livak method [24] using *recA* as an internal control. We also used *pvsA* as an alternate house-keeping gene for the mRNA expression analysis of competence related gene such as *tfoX*, *pilA*, *comEA* and the data were given as supplementary information.

### 2.7. Statistical Analysis

The data were analyzed by Student’s *t*-test. A probability level *(p)* value of ≤0.05 was considered statistically significant. Three independent experiments were done, and the data represents mean ± SE of these independent events.

## 3. Results

### 3.1. ChiS-Dependent Chitinase VPA0055 Is the Major Chitinase Required for Growth in the Presence of Chitin

**Figure 1:**
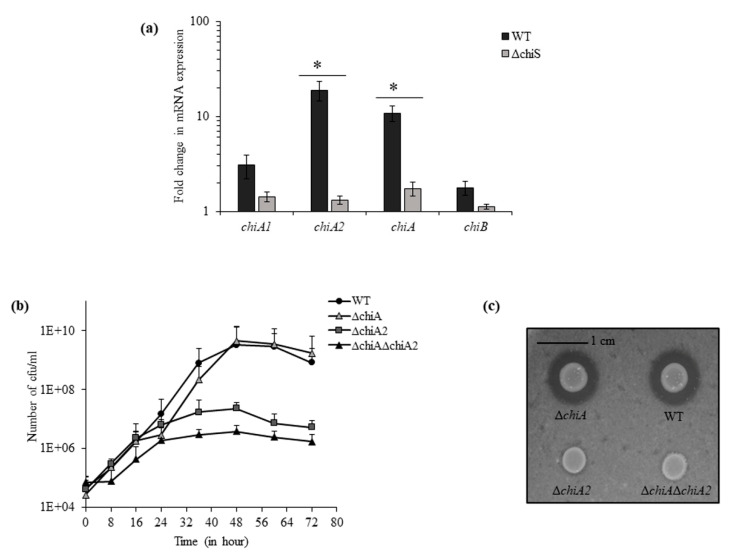
VPA0055 or ChiA2 is the major ChiS-dependent chitinase required for the growth of *V. parahaemolyticus* in the presence of chitin. (**a**) The relative expression of four chitinase genes at mRNA level was checked for the Δ*chiS* mutant compared to the WT strain. *recA* was used as an internal control. Each bar indicates the mean ± SE of three independent experiments. Asterisks represents *p* < 0.05, where a fold change in mRNA expression of the Δ*chiS* mutant was significantly affected. (**b**) Growth curve of the indicated isogenic mutants and WT in DASW with chitin as sole carbon source. Each point represents the mean ± SE of three independent experiments. (**c**) Chitinase plate assay. Wild type and indicated isogenic mutants of *V. parahaemolyticus* were assayed for chitinase activity using DASW plate containing 2% colloidal chitin.

The genes of chitin utilization pathway are known to be controlled by a sensor histidine kinase, ChiS in Vibrios [8]. In the presence of chitin flakes, the real-time RT-PCR showed 6-fold downregulation in the expression of chitinase gene *vpa1177* (chitinase A or *chiA*) and 14-fold downregulation in chitinase gene *vpa0055* (*chiA2*) in Δ*chiS* mutant as compared to WT. However, the expression of other two chitinase genes *vp0619* (*chiB*) and *vp2338* (*chiA1*) did not show any significant change between Δ*chiS* mutant and WT (Figure 1a).

Next, we used isogenic mutants Δ*chiA*, Δ*chiA2* and Δ*chiA*Δ*chiA2* for growth curve analysis compared to the WT strain in the presence of 1% chitin flakes. After 48 h, the maximum growth was observed and the viable count of WT, Δ*chiA*, Δ*chiA2* and Δ*chiA*Δ*chiA2* was 3.25 × 10^9^, 4.6 × 10^9^, 2.3 × 10^7^ and 3.8 × 10^6^ cfu mL^−1^, respectively (Figure 1b,c). So, the mutants Δ*chiA2* and Δ*chiA*Δ*chiA2* showed 140-fold and 850-fold reduced growth compared to the WT strain but the Δ*chiA* mutant showed WT-like growth. This suggests that in the absence of ChiA2, the activity of other three chitinase could support only minimal level of growth in chitin and thus it can be concluded that ChiA2 played the major role in the breakdown of chitin.

### 3.2. ChiA2 Is Essential for Chitin Induced Natural Transformation

**Figure 2:**
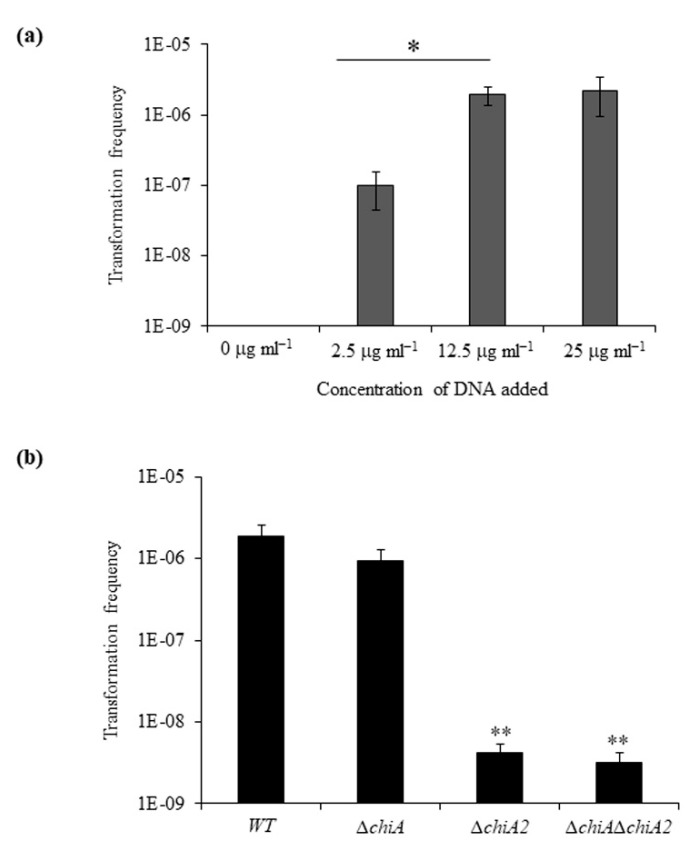
ChiA2 is essential for chitin-induced natural transformation (**a**) WT strain incubated with increasing concentration of extracellular DNA for natural transformation. (**b**) WT and isogenic mutant strains Δ*chiA*, Δ*chiA2* and Δ*chiA*Δ*chiA2* were naturally transformed on shrimp chitin flakes with 12.5 μg/mL extracellular DNA. Average of at least three independent experiments were represented. * Statistically significant difference between lowest and other concentrations of donor gDNA (*p* < 0.05); ** Statistically significant difference between the transformation frequency of WT and mutant strains (*p* < 0.05).

We tested the transformation frequency of WT strain in chitin flakes by adding increasing amounts of donor genomic DNA (gDNA) ranging from 0 to 25 µg mL^−1^ (Figure 2a). We observed increasing transformation frequencies of 1 × 10^−7^, 1.9 × 10^−6^ and 2.2 × 10^−6^ with gDNA concentration of 2.5 µg mL^−1^, 12.5 µg mL^−1^ and 25 µg mL^−1^, respectively. The frequency of spontaneous SmR-resistant mutants in a sample without DNA was below the limit of detection i.e., between 8 × 10^−10^ to 2 × 10^−10^. The transformation frequency difference between the addition of 2.5 µg mL^−1^ and 12.5 µg mL^−1^ was statistically significant.

Next, we compared the transformation frequency of WT, Δ*chiA*, Δ*chiA2* and Δ*chiA*Δ*chiA2*. The transformation efficiency of WT, Δ*chiA*, Δ*chiA2* and Δ*chiA*Δ*chiA2* was 1.89 × 10^−6^, 9.4 × 10^−7^, 4.2 × 10^−9^ and 3.2 × 10^−9^, respectively (Figure 2b). So, the mutants Δ*chiA*, Δ*chiA2* and Δ*chiA*Δ*chiA2* showed a 2-fold, 450-fold and 590-fold reduction in transformation frequency compared to the WT strain. Among the two ChiS regulated chitinases, the absence of ChiA2 drastically reduced the transformation frequency in *V. parahaemolyticus*. The transformation frequency difference between the WT and Δ*chiA* mutant was found to be statistically non-significant. The reason behind the high transformation frequencies of the Δ*chiA* mutant was the ability to utilize chitin efficiently. Therefore, like previous reports, this study also confirms that the ability to degrade chitin into soluble GlcNAc_n_ is directly linked to the DNA uptake efficiency [4,25].

### 3.3. Role of Transmembrane Regulators ChiS and TfoS in Natural Competence

**Figure 3:**
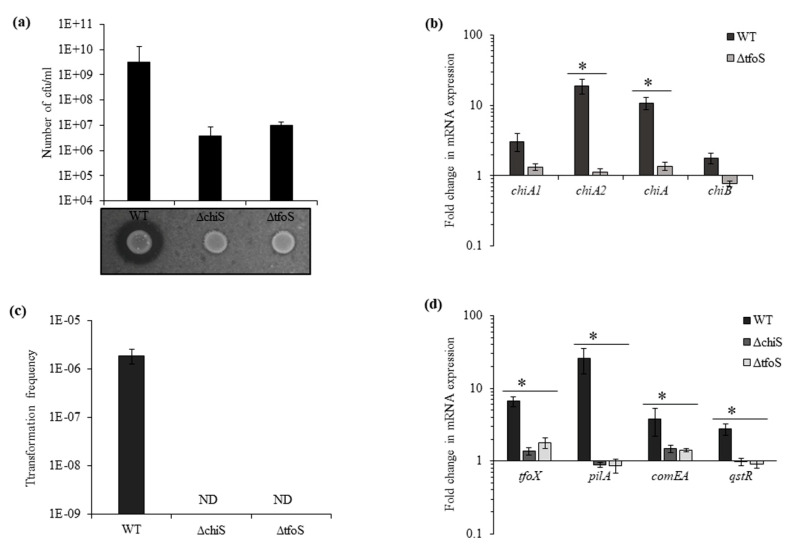
Transmembrane regulators ChiS and TfoS are essential for growth and natural transformation in the presence of chitin. (**a**) Growth of the indicated isogenic mutants and WT in DASW with chitin as sole carbon source after 48 h and chitinase activity on colloidal chitin plate. (**b**) The relative expression of four chitinase genes at mRNA level was checked for the Δ*tfoS* mutant compared to the WT strain. *recA* was used as an internal control. Each bar indicates the mean ±SE of three independent experiments. Asterisks represent *p* < 0.05. (**c**) WT and isogenic mutant strains Δ*chiS* and Δ*tfoS* were naturally transformed in the presence of shrimp chitin flakes. ND = not detected. (**d**) The relative expression of competence genes *tfoX*, *pilA*, *comEA* and *qstR* at mRNA level was checked for WT, Δ*chiS* and Δ*tfoS* mutant. Each bar indicates the mean ±SE of three independent experiments. Asterisks represents *p* < 0.05, where fold change in mRNA expression between wild type and mutant was significantly affected.

In addition to ChiS, TfoS was also depicted as a membrane-bound transcriptional regulator that links chitin sensing and induction of natural competence by activating TfoX [14,15] in *V. cholerae*. To determine the role of the homologous gene in *V. parahaemolyticus* in chitin utilization and natural transformation, we used isogenic mutants Δ*chiS* and Δ*tfoS*. The mutants were found to grow poorly in chitin as the sole carbon source, and the viable count for Δ*chiS* was 3.6 × 10^6^ cfu mL^−1^ and for Δ*tfoS* 9.7 × 10^6^ cfu mL^−1^ after 48 h (Figure 3a). The real-time RT-PCR indicates downregulation in the expression of chitinase genes in Δ*tfoS* mutant, *chiA2* showed 16-fold and *chiA* showed 8-fold downregulation (Figure 3b). This downregulation of chitinase genes in Δ*tfoS* mutant was similar to the Δ*chiS* mutant (Figure 1a).

In the presence of chitin, the natural transformation was undetectable in the Δ*chiS* and Δ*tfoS* mutants (Figure 3c). Along with downregulation in the expression of chitinases, Δ*chiS* and Δ*tfoS* mutants also showed a reduction in the mRNA expression of competence-related genes such as *tfoX*, *pilA*, *comEA* and *qstR* (Figure 3d and Figure S1). Therefore, it can be concluded that in the presence of chitin, the lack of chitinase activity in the Δ*chiS* and Δ*tfoS* mutant prevents the release of chitin oligosaccharides. Due to which, there was no upregulation in the expression of master competence regulator *tfoX* and other competence genes to induce the natural competence state in *V. parahaemolyticus*. Neither ChiS nor TfoS could independently upregulate the expression of *tfoX*.

### 3.4. ChiS and TfoS in Chitin Oligosaccharide Sensing

**Figure 4:**
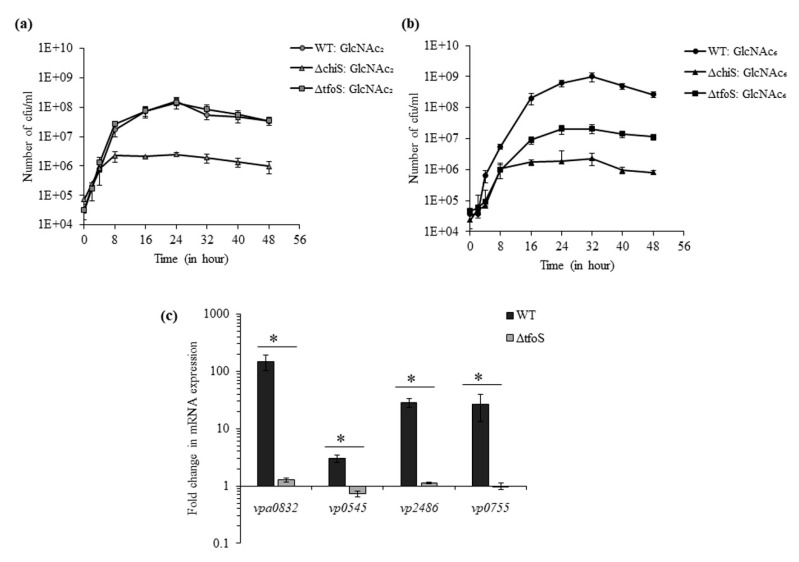
Role of transmembrane regulators ChiS and TfoS in chitin oligosaccharide sensing. (**a**) Growth curve of WT and isogenic mutant strains Δ*chiS* and Δ*tfoS* in 5 mM GlcNAc_2_ supplemented DASW for 48 h at 30 °C. (**b**) Growth curve of WT and isogenic mutant strains Δ*chiS* and Δ*tfoS* in 1.25mM GlcNAc_6_ supplemented DASW for 48 h at 30 °C. (**c**) The relative mRNA expression of the enzymes (*vpa0832*, *vp0545*, *vp2486* and *vp0755)* involved in the breakdown of GlcNAc_6_ was checked for WT and Δ*tfoS* mutant. Each bar indicates the mean ± SE of three independent experiments. * represents a *p*-value < 0.05.

We used the smallest chitin oligosaccharide, GlcNAc_2_, and the largest chitin oligosaccharide, GlcNAc_6_, for the growth analysis of WT, Δ*tfoS* and Δ*chiS*. In the presence of GlcNAc_2_, WT and Δ*tfoS* showed similar growth whereas Δ*chiS* mutant could not grow (Figure 4a). Therefore, it can be interpreted that the absence of *tfoS* did not inhibit GlcNAc_2_-induced ChiS-dependent activation of GlcNAc_2_ catabolic operon.

However, in the presence of GlcNAc_6_, both Δ*chiS* and Δ*tfoS* mutant showed reduction in growth compared to WT (Figure 4b). After 32 h, the viable count of WT, Δ*chiS*, Δ*tfoS* was 9.9 × 10^8^, 2.06 × 10^7^ and 2.3 × 10^6^ cfu mL^−1^, respectively (Figure 4c). So, the mutant Δ*tfoS* could grow just 10-fold more compared to the Δ*chiS* mutant. The Δ*chiS* mutant could not metabolize chitin oligosaccharides because GlcNAc_2_ catabolic operon was ChiS-dependent. As we already mentioned that GlcNAc_2_ catabolic operon was TfoS-independent, the reason behind reduced growth of the Δ*tfoS* mutant in GlcNAc_6_ might be related to the downstream processing of GlcNAC_6_ inside periplasm. The breakdown of GlcNAc_6_ inside the periplasm might depend on activities of chitodextrinase (*vpa0832:cdx*); N,N′-diacetylchitobiase (*vp0755: chb*) and two β-N-acetyl hexosaminidase (*vp2486: bNha* and *vp0545: ha*). Therefore, we compared the mRNA expression of these four enzymes in the presence of GlcNAc_6_ between the WT and Δ*tfoS* mutant. The Δ*tfoS* mutant showed 116.6-, 4.1-, 25.4- and 26.2-fold downregulation in *vpa0832*, *vp0545*, *vp2486* and *vp0755*, respectively (Figure 4c). So, the Δ*tfoS* mutant could not metabolize GlcNAC_6_ due to the downregulation of these four periplasmic enzymes.

### 3.5. ChiS and TfoS Are Indispensable for the GlcNAc_6_ Induced Competence State

**Figure 5:**
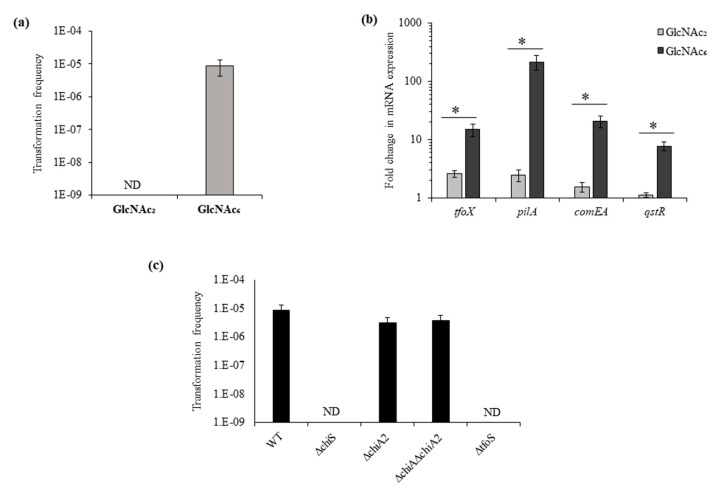
GlcNAc_6_ induced competence state in *V. parahaemolyticus* require ChiS as well as TfoS. (**a**) Induction of natural competence in WT in the presence of chitin oligosaccharides: 5 mM GlcNAc_2_ and 1.25 mM GlcNAc_6_ supplemented DASW. (**b**) The relative mRNA expression of competence genes *tfoX*, *pilA*, *comEA* and *qstR* was compared in the presence of GlcNAc_2_ and GlcNAc_6_ in the wild type strain. Each bar indicates the mean ±SE of three independent experiments. * represents a *p*-value < 0.05 (**c**) GlcNAc_6_ used for natural transformation in WT and indicated mutants. Each bar represents the mean ± SE of three independent experiments.

We found that GlcNAc_6_ could induce natural competence in the WT strain with a transformation frequency of 7.7 ± 3.6 × 10^−6^ (Figure 5a). It was reported that the periplasmic chitodextrinase could cleave GlcNAc_6_ into smaller molecules [6]. Therefore, we used GlcNAc_2_ as an inducer of natural transformation, but we could not detect transformants (below the limit of detection). So, we studied the mRNA expression of competence genes *tfoX*, *pilA*, *comEA* and *qstR* in the presence of GlcNAc_2_ and GlcNAc_6_. The GlcNAc_6_ induced upregulation as compared to GlcNAc_2_ was 5.7-, 87.5-, 13.6- and 6.9-fold for *tfoX*, *pilA*, *comEA* and *qstR*, respectively (Figure 5b and Figure S2). This showed that GlcNAc_6_ could upregulate the expression of these genes more significantly than GlcNAc_2_ (Figure 5b).

Then, we checked natural competence in the presence of GlcNAc_6_ for mutants which showed no transformation in the presence of chitin. In the growth medium of the Δ*chiA2* and Δ*chiA*Δ*chiA2* mutant when supplemented with purified GlcNAc_6_ instead of chitin, the mutant became competent and could uptake eDNA (Figure 5c). This result suggests that the reason behind the lack of competence in these two mutants was their inability to release GlcNAc_6_ moieties from chitin. Therefore, it can be concluded that ChiA2 plays a vital role in degradation of chitin to release GlcNAc_6_ residues which then induce a competence state. However, the Δ*chiS* and Δ*tfoS* mutants were unable to undergo transformation even in the presence of purified GlcNAc_6_ (Figure 5c). This indicates the inter-dependence of ChiS and TfoS because the absence of either lead to complete inhibition ofs GlcNAc_6_ induced competence in *V. parahaemolyticus*.

## 4. Discussion

In this study, we found that chitinase ChiA2 plays an essential role during growth on chitin as a sole carbon source and chitin-induced natural transformation. In the Δ*chiA2* mutant, the activity of three other chitinase (ChiA, ChiA1 and ChiB) could support only minimal level of growth (2.3 × 10^7^ cfu mL^−1^) which was just six-fold more when compared to the growth of Δ*chiS* mutant (3.8 × 10^6^ cfu mL^−1^). This suggests that chitinase VPA0055 (ChiA2) played the major role in breakdown of chitin in *V. parahaemolyticus*. This ChiS regulated chitinase, VPA0055 is 76.5% identical to VCA0027 (ChiA2) of *V. cholerae* and it was among the two major extracellular chitinases [4,25]. In addition to the major role played by ChiA2 in chitin-dependent growth, the activity of ChiA2 is critical for natural transformation in *V. parahaemolyticus*. Interestingly, the transformation frequency was drastically reduced in the Δ*chiA2* mutant, but not like Δ*chiS* or Δ*tfoS* mutant, where it was reduced beyond the detection limit. Similar observation was reported in *V. cholerae*, where absence of ChiA2 still support a low level of chitin-induced transformation due to other chitinases [25].

In *V. cholerae*, ChiS and TfoS were depicted as transmembrane regulators that link chitin sensing and the induction of natural competence by activating TfoX [13,14,15]. It was shown in *V. cholerae* that Δ*chiS* and Δ*tfoS* mutants were phenotypically different because Δ*chiS* could not utilize chitin oligomers, while a Δ*tfoS* mutant could [15]. However, in this study we found that the Δ*tfoS* mutant of *V. parahaemolyticus* showed a different growth phenotype depending on the size of the chitin oligosaccharide. In the presence of GlcNAc_2_, the growth phenotype of WT and Δ*tfoS* mutant was similar which suggests that, like *V. cholerae*, GlcNAc_2_ catabolic operon in *V. parahaemolyticus* is dependent on ChiS and independent of TfoS [14]. However, the inability of Δ*tfoS* mutant to utilize GlcNAc_6_ was found to be due to downregulation of chitodextrinase (*vpa0832*), N,N′-diacetylchitobiase (*vp0755*) and β-N-acetyl hexosaminidase (*vp0545* and *vp2486*). Therefore, Δ*tfoS* mutant could not utilize GlcNAc_6_ completely even in the presence of ChiS.

The induction of natural transformation with GlcNAc_6_ not by GlcNAc_2_ establishes GlcNAc_6_ as the key molecule required for this phenomenon. In addition, this might also suggest the presence of different sensors for the detection of GlcNAc_2_ and GlcNAc_6_. Because in the absence of *tfoS*, GlcNAc_2_ was able to activate ChiS-dependent GlcNAc_2_ catabolic operon. This observation proves ChiS as the sensor for the detection of GlcNAc_2_. The periplasmic sensor domain of TfoS is structurally related to hybrid two-component system (HTCS) proteins which can couple nutrient sensing to metabolic regulation [14,26]. It has been shown that the deletion of periplasmic domain of TfoS could abolish the natural transformation phenomenon in *V. cholerae* [15]. As we could detect natural transformation only in the presence of GlcNAc_6_, this might suggest a possible interaction between TfoS and GlcNAc_6_ either directly with the periplasmic loop or indirectly with the help of some unknown factor. So, TfoS might act as the sensor for GlcNAc_6_. However, none of them act independently because GlcNAc_6_ could not induce natural competence either in the Δ*chiS* or Δ*tfoS* mutant. Therefore, we speculate GlcNAc_2_ induced activation of ChiS and GlcNAc_6_ induced stimulation of TfoS; these two independent events might mutually control the transcriptional activation of *tfoR* and thus, the translation of master competence regulator TfoX.

Altogether, chitin-induced natural transformation is a common trait observed among Vibrio species, yet there are differences in the regulation of this phenomenon. A regulatory variation was observed by Simpson et al., where they mentioned quorum sensing is expendable for the natural transformation in *V. campbellii* DS40M4 and *V. parahaemolyticus* RIMD2201633 [16] but is required in *V. cholerae* to activate the competence regulator QstR. In this study, we found another variation where natural competence can only be induced by largest chitin oligosaccharide GlcNAc_6_ in *V. parahaemolyticus* whereas in *V. cholerae*, even smallest chitin oligosaccharide GlcNAc_2_ could induce the state of competence [14,15]. The significance of GlcNAc_6_-dependent natural transformation lies in the fact that GlcNAc_6_ makes this process more specific for Vibrios because relatively few other microbes can take up long chitin oligosaccharides compared to mono- and di-saccharides [27] and that ultimately leads to the acquisition of new features. As a consequence, novel strains of Vibrios could emerge with heightened ecological fitness and pathogenicity [28]. Moreover, in a nutrient-poor marine environment, the ability to uptake GlcNAc_6_ might provide them a competitive advantage over other microbial species. In future, we would like to know whether GlcNAc_6_-dependent competence is a strain specific phenomenon or a general trait for *V. parahaemolyticus* and more detailed genetic analysis will be done to elaborate GlcNAc_6_ induced competence in *V. parahaemolyticus*.

## Figures and Tables

**Table 1:** List of strains and plasmids used in this study.

Strains or Plasmids	Description	Source
Strains		
*V. parahaemolyticus* **RIMD22106333**	Clinical isolate of serotype O3:K6; wild-type (WT) strain	[1]
*VPS*	Spontaneous streptomycin-resistant (SmR) mutant	
Δ*chiS*	Deletion in *vp2478* of WT, sensor kinase	This study
Δ*chiA*	Deletion in *vpa1177* of WT, Chitinase A	This study
Δc*hiA2*	Deletion in *vpa0055* of WT, chitinase	This study
Δ*chiA*Δ*chiA2*	Double mutant	This study
Δ*tfoS*	Deletion in *vp0854* of WT, AraC family transcriptional regulator	This study
***Escherichia coli***		
β2155	*thrB1004 pro thi strA hsdS* Δ(*lacZ*)ΔM15 [F′ Δ(*lacZ*)M15 *lacI*^q^ *traD36 proA*^+^*proB*^+^] Δ*dapA*::*erm*(Em^r^), *pir*::RP4; *kan*(Km^r^) from SM10	[19]
JM109	*rec*A1 *end*A1 *gyr*A96 *thi hsd*R17 (rK−,mK+) *rel*A1 *sup*E44 ∆(*lac-pro*AB) [F′, *tra*D36, *pro*AB, *lac*IqZ∆M15]	Promega
**Plasmids**		
pGEM-T easy	TA-cloning vector; Amp^R^	Promega
pXAC623	Suicide vector derived from pKTN701 containing the *sacB*gene of *Bacillus subtilis*; Cm^R^	[18]

Amp^R^, ampicillin-resistant; Cm^R^, chloramphenicol-resistant; SmR, streptomycin-resistant.

## Supplementary Materials

The following are available online at https://www.mdpi.com/2076-2607/8/9/1303/s1, Figure S1: The relative expression of competence genes *tfoX*, *pilA*, *comEA* and *qstR* at mRNA level was checked for WT, Δ*chiS* and Δ*tfoS* mutant in the presence of chitin; Figure S2: The relative mRNA expression of competence genes *tfoX*, *pilA*, *comEA* and *qstR* was compared in the presence of GlcNAc_2_ and GlcNAc_6_ in wild type strain.

## Abbreviations

MDPI	Multidisciplinary Digital Publishing Institute
DOAJ	Directory of open access journals
TLA	Three letter acronym
LD	linear dichroism
TfoS	ChiS-dependent *tfoR* regulator
DASW	Defined artificial sea water
GlcNAc	N-acetyl glucosamine
GlcNAc_2_	Diacetylchitobiose
GlcNAc_6_	Hexaacetylchitohexaose
