# Supplementary material for: Regulation of Chitin-Dependent Growth and Natural Competence in Vibrio parahaemolyticus
Source: Microorganisms. 2020 Aug 26;8(9):1303. doi: 10.3390/microorganisms8091303 (PMC7564644; doi:10.3390/microorganisms8091303)
Supplement: Supplementary file 1 [file microorganisms-08-01303-s001.pdf]

Manuscript No.: microorganisms-889985

Regulation of Chitin Dependent Growth and Natural Competence in *Vibrio parahaemolyticus*

ANUSUYA DEBNATH[1*], TAMAKI MIZUNO[1] and SHIN-ICHI MIYOSHI[1]

[1]Okayama University, Okayama, Japan

***Corresponding author**

Anusuya Debnath

Graduate School of Medicine, Dentistry and Pharmaceutical Sciences

Okayama University

1-1-1, Tsushimanaka, Kita-ku

Okayama 700-8530

Phone: +81-86-251-7959

e-mail: anusuyadebnath@yahoo.co.in, pu0l39jq@okayama-u.ac.jp

Supplementary Figures

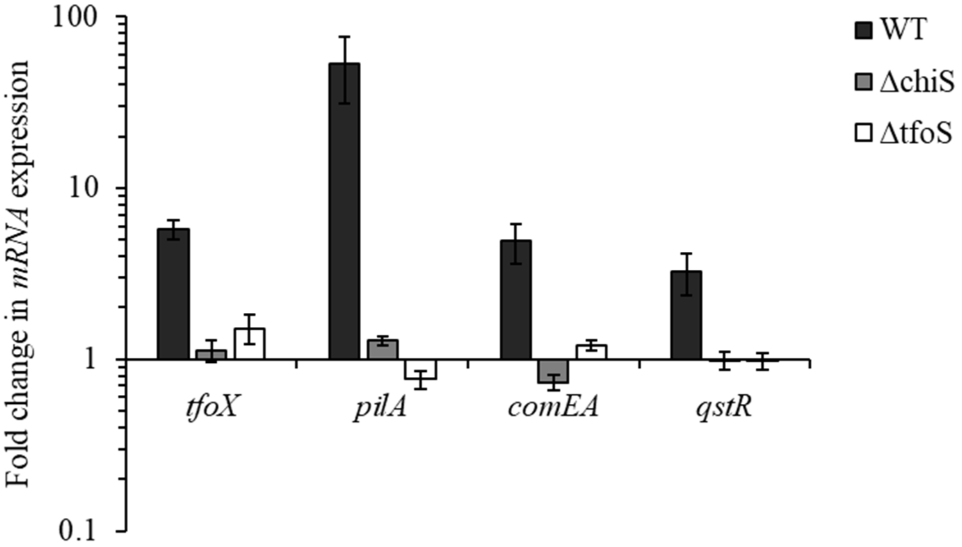

Figure S1. The relative expression of competence genes *tfoX*, *pilA*, *comEA* and *qstR* at mRNA level was checked for WT, Δ*chiS* and Δ*tfoS* mutant in the presence of chitin. *pvsA* was used as an internal control. Each bar indicates the mean ± SE of three independent experiments.

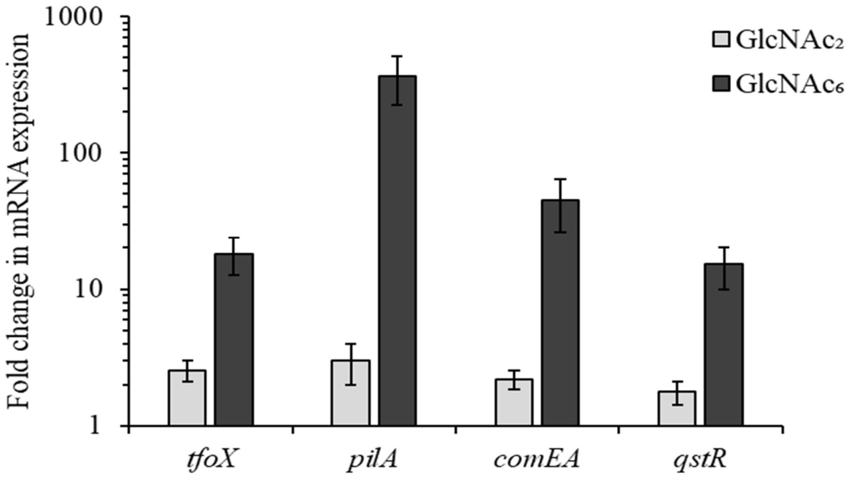

Figure S2. The relative mRNA expression of competence genes *tfoX*, *pilA*, *comEA* and *qstR* was compared in the presence of $GlcNAc_2$ and $GlcNAc_6$ in wild type strain. *pvsA* was used as an internal control. Each bar indicates the mean ± SE of three independent experiments.
